# Knowledge transfer of eLearning objects: Lessons learned from an intercontinental capacity building project

**DOI:** 10.1371/journal.pone.0274771

**Published:** 2022-09-20

**Authors:** Hooi Min Lim, Chirk Jenn Ng, Heather Wharrad, Yew Kong Lee, Chin Hai Teo, Ping Yein Lee, Kuhan Krishnan, Zahiruddin Fitri Abu Hassan, Phelim Voon Chen Yong, Wei Hsum Yap, Renukha Sellappans, Enna Ayub, Nurhanim Hassan, Sazlina Shariff Ghazali, Puteri Shanaz Jahn Kassim, Nurul Amelina Nasharuddin, Faridah Idris, Michael Taylor, Cherry Poussa, Klas Karlgren, Natalia Stathakarou, Petter Mordt, Stathis Konstantinidis

**Affiliations:** 1 Department of Primary Care Medicine, Faculty of Medicine, Universiti Malaya, Kuala Lumpur, Malaysia; 2 Department of Research, SingHealth Polyclinics, Singapore, Singapore; 3 Duke-NUS Medical School, Singapore, Singapore; 4 School of Health Sciences, University of Nottingham, Nottingham, England; 5 UM eHealth Unit, Faculty of Medicine, Universiti Malaya, Kuala Lumpur, Malaysia; 6 Dean’s Office, Faculty of Medicine, Universiti Malaya, Kuala Lumpur, Malaysia; 7 Department of Building Surveying, Faculty of Built Environment, Universiti Malaya, Kuala Lumpur, Malaysia; 8 School of Biosciences, Faculty of Health & Medical Sciences, Taylor’s University, Subang Jaya, Selangor, Malaysia; 9 School of Pharmacy, Faculty of Health & Medical Sciences, Taylor’s University, Subang Jaya, Selangor, Malaysia; 10 Taylor’s Digital, Taylor’s University, Subang Jaya, Selangor, Malaysia; 11 Teaching and Educational Development (TED), Centre of Future Learning, Taylor’s University, Subang Jaya, Selangor, Malaysia; 12 Department of Family Medicine, Faculty of Medicine and Health Sciences, Universiti Putra Malaysia, Serdang, Malaysia; 13 Department of Multimedia, Faculty of Computer Science and Information Technology, Universiti Putra Malaysia, Serdang, Malaysia; 14 Department of Pathology, Faculty of Medicine and Health Sciences, Universiti Putra Malaysia, Serdang, Malaysia; 15 Department of Learning, Informatics, Management and Ethics (LIME), Karolinska Institutet, Stockholm, Sweden; 16 NettOp, Department of E-Learning Development, University of Stavanger, Stavanger, Norway; Universiti Kebangsaan Malaysia Fakulti Perubatan, MALAYSIA

## Abstract

**Background:**

Effective knowledge transfer of eLearning objects can hasten the adoption and dissemination of technology in teaching and learning. However, challenges exist which hinder inter-organisational knowledge transfer, particularly across continents. The ACoRD project aimed to transfer knowledge on digital learning development from UK/EU (provider) to Malaysian (receiver) higher education institutions (HEIs). This study explores the challenges encountered during the knowledge transfer process and lessons learned.

**Methods:**

This is a qualitative study involving both the knowledge providers and receivers in focus group discussions (n = 25). Four focus group discussions were conducted in the early (n = 2) and mid-phase (n = 2) of the project by trained qualitative researchers using a topic guide designed to explore experiences and activities representing knowledge transfer in multi-institutional and multi-cultural settings. The interviews were audio-recorded, transcribed verbatim, and checked. The transcripts were analysed using thematic analysis.

**Results:**

Five main themes emerged from this qualitative study: mismatched expectations between providers and receivers; acquiring new knowledge beyond the professional "comfort zone"; challenges in cascading newly acquired knowledge to colleagues and management; individual and organisational cultural differences; and disruption of knowledge transfer during the COVID-19 pandemic.

**Conclusion:**

This study highlights the need to create a conducive platform to facilitate continuous, timely and bi-directional needs assessment and feedback; this should be done in the early phase of the knowledge transfer process. The challenges and strategies identified in this study could guide more effective knowledge transfer between organisations and countries.

## Introduction

During the COVID-19 pandemic, higher education institutions (HEIs) faced challenges delivering educational activities due to the forced global shutdown. Digital learning has become a crisis-response strategy; however, the universities, faculty, and students face challenges in digital learning due to a lack of skills, competency, availability, resources, and implementation strategy [1]. HEIs from low-middle income countries (LMICs) experienced a greater impact as digital learning technology and programs are yet to be established [2]. There is increased attention on digital transformation in the HEIs to adequately prepare, develop and apply the digital technology optimally in educational activities [3]. Therefore, capacity building of digital learning in HEIs is essential to create an effective online teaching and learning environment in LMICs.

Instead of reinventing the wheel in exploring strategies to improve digital learning in HEIs, knowledge transfer of skills and technology from experienced HEIs would potentially hasten the process of capacity building and fully utilise the limited funding available in LMICs. Knowledge transfer is a process by which knowledge of making useful things contained within one organization is brought into use within another organizational context [4]. Knowledge transfer between organisations could foster capacity development, reduce redundancy, and leverage the knowledge bases in LMICs [5]. However, inter-organisational knowledge transfer is challenging and influenced by the organisations’ knowledge, organisational, and network characteristics [6]. Though research has been conducted looking at individual-level knowledge sharing and knowledge transfer between academia and industry [7, 8], there is a lack of research on how to effectively conduct the process of knowledge transfer between HEIs for capacity building.

Cross-sector and interprofessional collaboration is recommended as a model for digital learning development. However, challenges such as time constraints, synchronisation of members’ availability, funding, communication between developers and content authors, and members’ commitment were identified in such collaborations [9]. The collaboration would be more complex and challenging when involving the transfer of knowledge across different professions from different HEIs. Connolly et al. [10] point to some challenges in knowledge transfer among HEIs in digital learning development, such as management issues, organisational differences, and working relationships. Therefore, exploring the challenges in the process of knowledge transfer, especially across HEIs from different geographical locations and cultural backgrounds, are helpful in developing strategies to foster collaboration.

In this study, we aim to explore the challenges of knowledge transfer between HEIs i.e. UK/EU/Malaysian universities, using an eLearning object development project as a case study [11]. Based on these challenges, we hope to propose strategies that could optimise international knowledge transfer to foster the capacity building of digital learning in LMICs. This study would add value to the existing literature on knowledge management in digital learning development between HEIs.

## Methods

### The context: ACoRD project

The Advancing Co-creation of RLOs to Digitise Healthcare Curricula (ACoRD) is a European Union (Erasmus+) funded 3-year project with the collaboration of six institutions from the United Kingdom, Sweden, Norway, and Malaysia, i.e., University of Nottingham (UoN), University of Stavanger (UiS), Karolinska Institute (KI), Universiti Malaya (UM), Universiti Putra Malaysia (UPM) and Taylor’s University (TU). The project aims to introduce innovative digital pedagogy methods to benefit healthcare and biomedical science students in Malaysia. This was achieved by developing high-quality, peer-reviewed multimedia digital tools and resources and integrating them into existing curricula [12]. UoN, UiS, and KI are centres of excellence for e-learning in Europe that have a strong foundation, experiences, and teams in producing high-quality reusable e-learning materials. The Health eLearning and Media (HELM) team from UoN has pioneered the design and development of reusable learning objects (RLOs) in healthcare and education for the past 20 years [13]. In this ACoRD project, UK/EU partners aimed to cascade their pedagogical, technical, and design skills in building RLOs to the Malaysian institutions. Although the three Malaysian partners have prior experience in developing digital learning materials, the development process is ad hoc and unstandardised. The digital learning teams in Malaysian HEIs were less established with a lack of technical and media development expertise, particularly in relation to digital pedagogy and design methodology. The ACoRD team consists of healthcare educators, eLearning experts, and learning technologists. The ACoRD project aims for the UK/EU academic institutions to transfer their knowledge and experiences in developing RLOs and eLearning to the Malaysian institutions using a co-creation methodology called ASPIRE (Aim, Storyboarding, Populating, Implementing, Release, Evaluation) which includes the following phases [14]:

- Aims: organise stakeholder workshops, train-the-trainer workshops, training workshops in Malaysian partners’ institutions.
- Storyboarding: internal storyboard workshops involving educators and students.
- Population: writing and review of the specification.
- Implementation: technical creation of RLOs.
- Release: release of RLOs into target population, focus on the actual incorporation of RLO resources into the curriculum of the health sciences programmes, creation of open access repository to host the RLOs created.
- Evaluation: Evaluate the quality of all phases of the projects, the effectiveness and usage of RLO.

### Study design

A qualitative methodology was used to explore the views and experiences of ACoRD project participants about the knowledge transfer process. Focus group discussions were conducted to interview the project participants, encouraging interaction between participants and generating ideas. Focus group discussions gathered a wider range of opinions and insights from ACoRD participants on their experiences, challenges, and lessons learned in the knowledge transfer process.

### Theoretical framework

**Fig 1:**
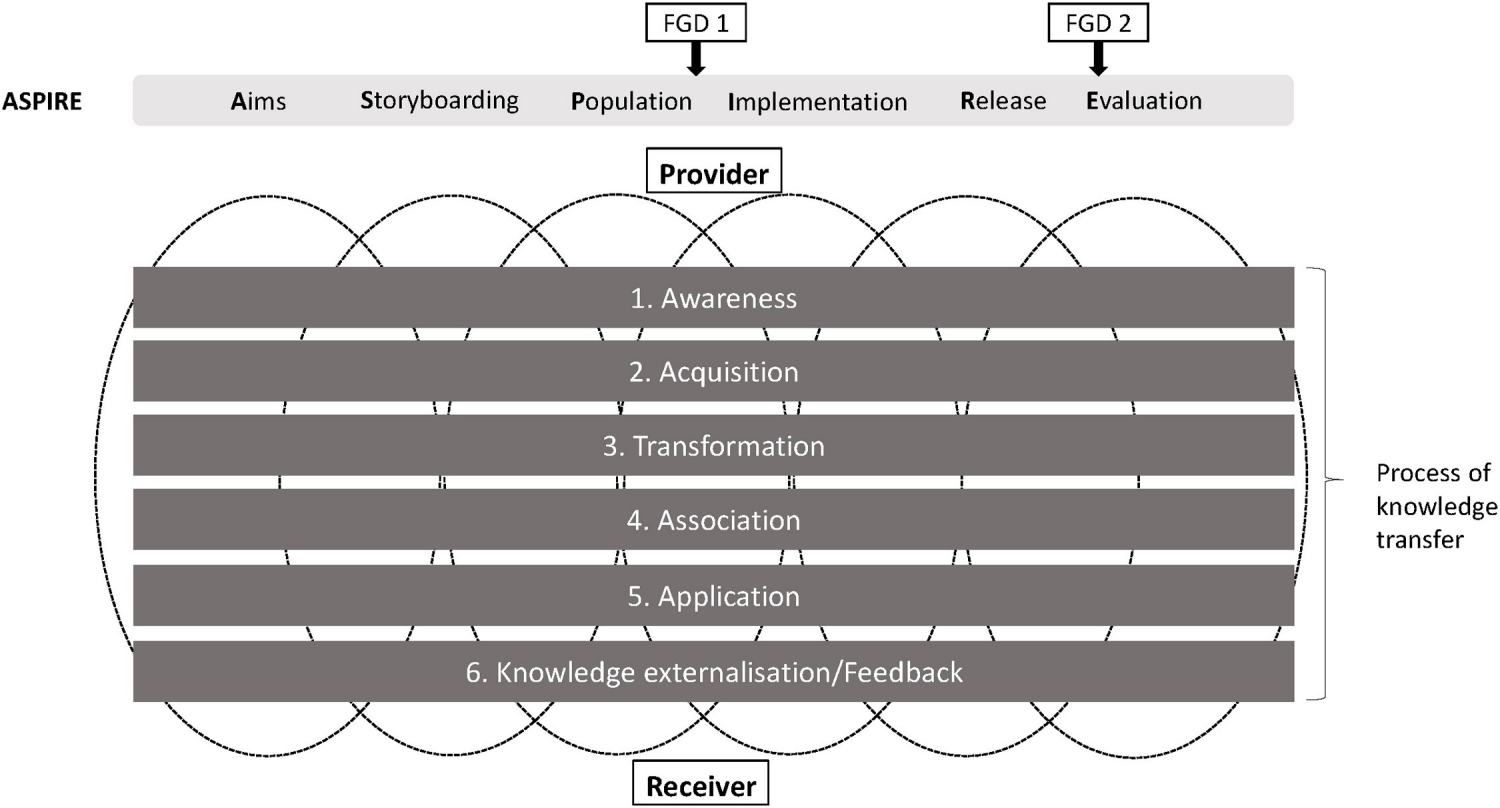
Theoretical framework for knowledge transfer of ASPIRE, a methodology for developing reusable learning objects, between UK/EU (provider) and Malaysian (receiver) universities. Adapted from Liyanage et al. (2009). FGD, focus group discussion.

A knowledge transfer model proposed by Liyanage et al. [15] was used as the theoretical framework to develop the topic guide for this interview. In this model, knowledge transfer consists of six main processes: awareness, acquisition, transformation, association, application, and feedback [15]. In addition, we have included the ASPIRE methodology into the theoretical framework because each knowledge component within the ASPIRE methodology would have gone through the same knowledge transfer process along the project time frame, which followed the steps in ASPIRE (Fig 1). The topic guide is available in S1 File.

### Study participants and setting

Four focus group discussions were conducted sequentially during the timeline of this project. Two focus group discussions were conducted face-to-face during the project workshop in December 2019 in Malaysia. Two other focus group discussions were conducted virtually in October 2020 via the Zoom platform during the virtual project workshop due to the COVID-19 pandemic. The study participants included were the ACoRD project participants from six partner universities who had been actively involved in the ACoRD project’s activities and RLO development.

### Data collection

The data collection was conducted at two-time points along with the ASPIRE framework. The first two focus group discussions were conducted in December 2019 when the progress of the ACoRD project was at the “A”, “S” and “P” phases of “ASPIRE framework”. The other two focus group discussions were conducted in October 2020 when the progress of the ACoRD project was at the “I”, “R”, “E” of phases of “ASPIRE framework”.

Ethics approval was obtained from the Universiti Malaya Medical Centre Medical Research Ethics Committee (MREC number 2019108–7914) before the data collection. Purposive sampling was used to select the participants based on: (1) universities from six partners and (2) participants’ roles in the ACoRD project (e.g., project manager, instructional designer, learning technologist, researcher, and educator). An invitation to participate in FGDs was sent through email before the workshops. Participants read through the participant information sheet, and written consent was obtained from those willing to participate. All participants filled up a demographic data sheet before the FGDs.

During the FGDs, participants from three Malaysian universities (receivers) were grouped into the same group, while participants from the UK and European universities (providers) were grouped into another group. The aim of FGD grouping according to the provider/receiver role was to encourage open discussions among the participants who have the same role in the knowledge transfer process.

There were 8–10 participants in each FGD which lasted approximately one hour. Each FGD was voice-recorded using audio recorders. The participants gave their consent to recording the FGD. The FGD was conducted by two researchers (CJN and HW) who were experienced in conducting qualitative research and FGDs. CJN, the Malaysian universities’ project leader, interviewed the UK/EU group (provider). HW, the UK/EU universities’ project leader, interviewed the Malaysian group (receiver). The interviews were transcribed verbatim. The names of the participants and institutions were anonymised. Data collection stopped when thematic saturation was reached.

### Data analysis

All voice recordings were transcribed verbatim and checked independently by a researcher (HML) for accuracy. Field notes taken during the FGDs were used to supplement the analysis. HML completed all the coding, and CJN double-coded one transcript to ensure consistency. Two main researchers (HML and CJN) met regularly to discuss the coding from the transcripts. Any coding discrepancy was resolved through discussion with members of the research team (HML, CJN, HW, YKL, and CHT) until a consensus was reached. We used a thematic analysis method for this qualitative data analysis [16]. Open codes were assigned for each quotation, and the codes were systematically categorised to form a coding framework. Subsequently, we identified the themes that emerged from the data. Quotes most representative of the essence of the themes were extracted for presentation in this manuscript. Coding and analysis of the transcripts were performed using Atlas.it version 8.

Two researchers (HML and CJN), who were directly involved in the coding and thematic analysis, consciously detached from their roles in the ACoRD project during data analysis. CJN was the lead for the Malaysian HEIs, while HML was one of the content authors in the Universiti Malaya team. We constantly reflected on our roles in the ACoRD project and our potential bias throughout all phases of data analysis. The emerging themes were discussed, and the consensus was reached with two other researchers, HW (the ACoRD lead from UK/EU HEIs) and YKL (an academician with experience with digital learning development). The names of the interviewees were anonymised by an independent research assistant who did not participate in this qualitative research.

## Results

### Participants’ demographic profile

**Fig 2:**
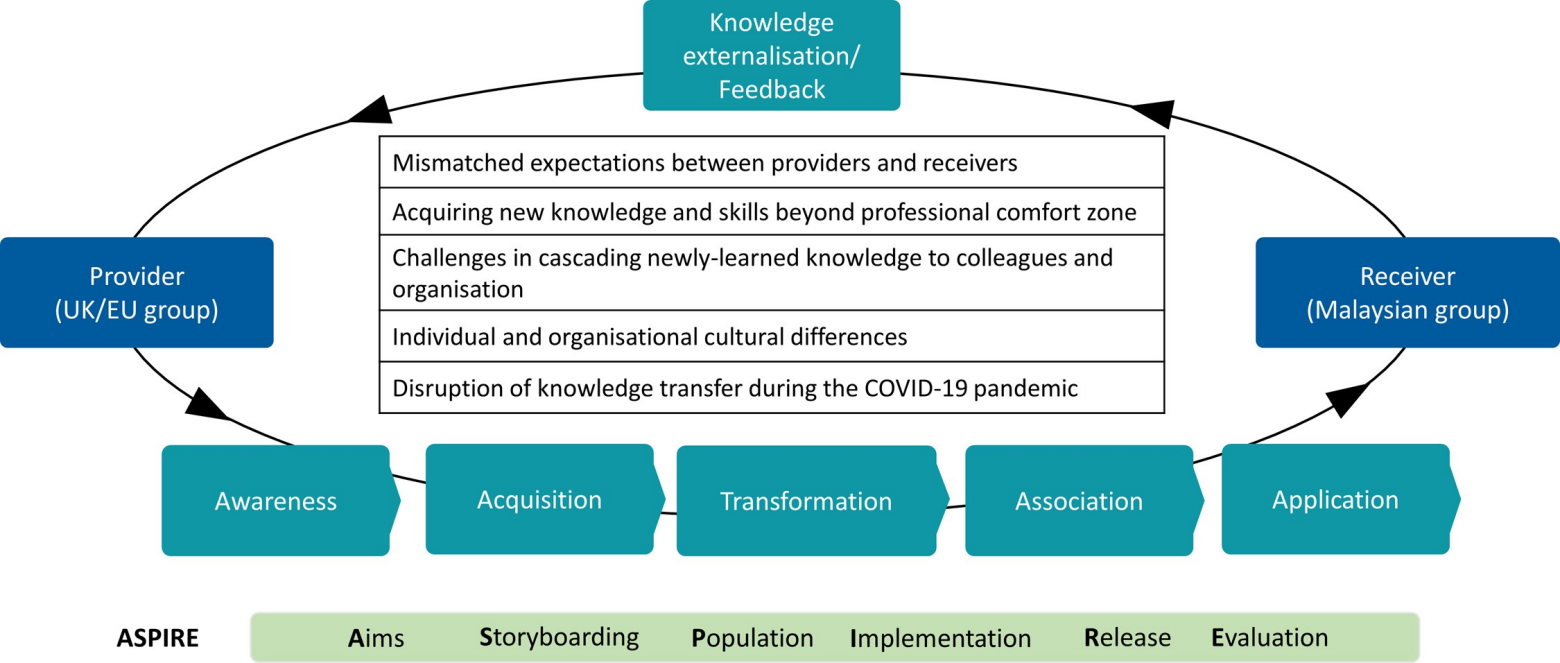
Challenges related to knowledge transfer of the ASPIRE framework.

A total of 25 participants were recruited for the FGDs: 17 participants from the Malaysian universities (receiver) and 9 participants from the UK/EU universities (provider). Among them, there were institutional leaders (n = 4), project managers (n = 4), instructional designers (n = 6), learning technologists (n = 5), medical educators (n = 3), and project research assistants (n = 3). The years of experience in eLearning ranged from 1–25 years. Five themes on the challenges related to the knowledge transfer of the ASPIRE eLearning object development methodology emerged from the analysis (Fig 2).

### Theme 1: Mismatched expectations between providers and receivers

Both providers and receivers expressed the mismatch of expectations on the scope and depth of knowledge to be transferred, especially the transfer of technical skills. Three subthemes emerged under this theme.

#### Subtheme 1.1: Mismatched on the types and levels of technical skill transfer

Providers and receivers had different expectations and opinions on the types and levels of technical skills to be transferred. Providers expressed that receivers’ technical teams expected a higher level of technical skills training, where they had difficulty meeting the expectations. It was a challenge for providers to transfer the knowledge accumulated over the years to receivers within the limited time frame of a project.

*“…in three days training*, *while it usually takes like 3–4 years to do that… I think it was really difficult to manage the expectations there…” (P7*, *provider*, *project manager)*

From the receivers’ perspective, they were keen to learn more in-depth about the processes, especially transforming the specification into the end-product, which were not guided in detail by the providers.

*“…They (providers) introduced some of the software*, *but they don’t actually explain how*, *from the specification itself*, *and how it is translated into the end products*.*” (P9*, *receiver*, *instructional designer)*

There was a mismatch in the expectations on the depth of technical skills to be transferred. Receivers preferred to learn the overall process of developing the technical components of RLOs. As one receiver described:

*“I feel that trainer goes too much into how to edit graphic*, *how to edit video*… *I think we can learn that on our own*. *But it’s more like how the process from the specifications*, *and the creation*, *we just want to know the steps*, *not how to edit using software…It’s more on how to do in a systematic way from the specifications onward*.*” (P9*, *receiver*, *instructional designer)*

#### Subtheme 1.2: Variation of technical skills among receivers

Providers expressed that they faced difficulty delivering the knowledge and skills at receivers’ expectations because of a wide variation of baseline technical skills among the receivers. Responses from the providers showed that it was challenging to pitch at the right scope and depth of training as it depends on the individual expertise and previous experience in eLearning objects development.

*“It was a challenge to know which level to put the training on*, *the level of expertise…so you could go in depth*, *but it will require them to have some previous knowledge*‥*all these challenges in groups like this*, *you have some others that know a lot; some other person that hasn’t seen the programme before*, *so it was difficult…*.*” (P16*, *provider*, *learning technologist)*

Though the providers had evaluated the receivers’ technical skills before the training sessions, it was difficult to plan the training and accommodate all the receivers’ needs. One of the providers stated that:

*“…when it goes down to the planning of the training*, *so you got the list with skills that the people had*, *to build upon those…some people that know a lot about one topic and very little about the other*. *So*, *it was really difficult to accommodate that*, *actually build the existing skills*‥*” (P7*, *provider*, *project manager)*

To address the challenge, the providers conducted an assessment of the existing resources in receivers’ institutions, their needs, and requirements regarding eLearning software and equipment. The providers tailored the technical training according to the receivers’ technical ability. A technical consultation platform between receivers and providers was set up to ease the process of inquiry and solve technical problems.

*“I think the first visit out to Malaysia was very important actually*, *setting the scene engaging competencies*, *and engaging needs*, *the technical requirements*, *support and things like that*.*” (P5*, *provider*, *project manager)*

*“[Provider’s technologist] helped us to make a great preparation for the technical workshop*, *because he helps to prepare material for different levels*. *They [provider’s technologists] were starting with the simplest level*, *just to make sure that everything is understood by everyone*. *But at the same time*, *they have this toolkit of other examples that can go into more complicated level*, *if needed*.*” (P7*, *provider*, *project manager)*

*“[Receiver’s technologist] converse with [Provider’s technologists] to get their help*, *especially when there is a bug*, *or trouble somewhere in the page*. *And I think [provider’s technologist] responded*, *and I think he sort of taught us as well*.*” (P6*, *receiver*, *project manager)*

#### Subtheme 1.3: Bidirectional feedback to address mismatched expectations

Both providers and receivers found that the bidirectional feedback was useful to solve the mismatch of expectations in the process of knowledge transfer. The receivers gave feedback to the providers on their learning needs and requested specific technical knowledge to be transferred after the first training session.

*“…I think all of us agree that it (training session) was well conducted and we learned a lot*. *And then*, *in June again we had more training in terms of technical as we requested UK/EU partners to give the technical part of it…it has been done very well*.*” (P6*, *receiver*, *project manager)*

Providers agreed on the importance of feedback from receivers and suggested that continuous assessment of the learning needs might help improve the knowledge transfer process. For example, one of the providers said:

*“…probably we should take a step back and do small assessments within the training…take 15 minutes to receive the feedback whether the information has reached the receiver*. *So we can have like go back and forth within the training*.*” (P7*, *provider*, *project manager)*

Providers described their strategies for giving feedback to support the receivers during the knowledge transfer process. They tried to provide balanced feedback and avoided being overly critical of the receivers’ work to keep them motivated.

*“…you have to be really careful when you are giving the feedback because you don’t want to overdo it… And…um…give the impression to the people who are receiving it that they are doing something wrong… when you are criticizing too much*, *you might demotivate the people*. *Sometimes you have to balance what’s more important*, *keep a really good communication…*.*” (P7*, *provider*, *project manager)*

### Theme 2: Acquiring new knowledge and skills beyond professional "comfort zone"

Participants experienced challenges when transferring knowledge across different professions. Three subthemes emerged under this theme.

#### Subtheme 2.1: Personal challenges faced by receivers from different professions

The learning technologists (receivers) were concerned about the time and effort needed for the development of RLOs in this project, especially when their institutions did not have an established eLearning technical team.

*“…We are not actually dedicated our time just for this project…It’s quite challenging to present it to the management… we need to be creative in how we align with the final goal and make sure our own team is not exhausted*.*” (P9*, *receiver*, *instructional designer)*

Educators (receivers) struggled to implement the activities and ideas from the storyboards into the specification because of technical constraints such as manpower, technical resources, and time. As such, educators had to lower their expectations to match what was feasible for technical development.

*“…when we did the storyboarding*, *it sounds all very exciting because the students came up with so many ideas*. *And then when the lecturer is at the specification writing in*, *consultation with the technical team*, *he had to tone it down*, *because that would require a lot more manpower*, *resources*, *time*, *etc*. *So we had to tone it down to what is feasible…” (P12*, *receiver*, *educator)*

#### Subtheme 2.2: Challenges in understanding each other’s work

Learning technologists in the providers’ team had difficulty understanding academic work such as needs assessment, evaluation, and educational research on eLearning. However, they considered it as a good opportunity to be involved in academic work.

*“… my personal biggest challenge was that the fact I came from the technical department at our university*, *so I kind of like having a hard time figuring for the academic side of the project and keeping track… it’s a challenge*, *because that’s not part of my daily work… but it is a good challenge*…*” (P3*, *provider*, *learning technologist)*

The providers’ technical team also expressed that they were not used to teaching a large amount of knowledge and materials required by this project. Therefore, they found it challenging to plan the training and teach for long hours. However, they had positive attitudes and considered it a great opportunity to expand their capacity in teaching and research.

*“… that was very interesting*, *very challenging*, *extremely challenging*, *and you know I’m a techy*, *I’m not used to and had given training before*, *but to do whole day and plan it*.*” (P15*, *provider*, *learning technologist)*

*“… I learnt a lot and it is interesting*, *but I haven’t had much expertise in it (teaching)*, *I guess I could be much better at it…" (P16*, *provider*, *learning technologist)*

Likewise, the academics from the receivers group had difficulty understanding the technical development process of eLearning at the initial phase of the project. Worries arose among the receivers regarding coding and technical development. One of the academicians said:

*“…the main part that we are worried about is all the coding and all that*. *I think initially we were like thinking if need to do all the hard coding so it will very very difficult for us…" (P2*, *receiver*, *institutional lead)*

#### Subtheme 2.3: Positive and safe environment for collaboration

Despite difficulties in transferring new knowledge, providers described that the receivers had positive attitudes and great motivation to learn new knowledge. *“I think that somehow there is a higher motivation which I cannot explain how it has been achieved" (P8*, *provider*, *project manager)*. In addition, the receivers were enthusiastic about acquiring new knowledge, "*enthusiasm among the partners in Malaysia" (P1*, *provider*, *institutional lead)*, and were willing to accept suggestions from the providers.

*I realised that they are very willing to learn*, *so they were like listening to what we were saying*, *when we were proposing*, *and they were asking questions… I didn’t feel the kind of…err… negative vibes… the questions they were always targeting to the point*, *and then they were willing to learn*. *(P19*, *provider*, *learning technologist)*

Likewise, receivers expressed that the providers were very helpful and responsive to their questions and requests. One of the receivers said, *“So far the European team has been very helpful*, *when we ask you give*, *we ask you give*.*” (P6*, *receiver*, *project manager)* The providers were willing to share their knowledge and resources with the receivers to facilitate their RLOs development.

*“…provide all the knowledge that we have*, *not fearing that other people are going to steal my work*,*…I believe it is a win-win situation because through the perspective of the European partners that transferring the work…erm…somehow the impact of your work expands beyond the narrow border of your country or your continent…*.*” (P7*, *provider*, *project manager)*

### Theme 3: Challenges in cascading newly-learned knowledge to colleagues and organisation

#### Subtheme 3.1: Incremental confidence in cascading the knowledge

At the beginning of the project, receivers lacked confidence in cascading the newly-learned knowledge to their colleagues in respective institutions, in line with a train-the-trainer approach, especially when they had no experience in developing RLOs. The receivers described:

*“During the workshop*, we are supposed to teach the rest (their colleagues) using the online specifications (new tool provided by the providers)*everyone chuckles* …it was very challenging… as if we don’t know what to do.*” (P20*, *receiver*, *educator)*

*“*‥*My confidence level is not very good at that point of time (during the training workshop to their colleagues)…even though we have done the storyboarding at UK…" (P22*, *receiver*, *educator)*

However, the confidence level in cascading the knowledge increased after receivers had gone through the whole developmental process of RLOs and organised several internal workshops within their own institutions.

*“I’m not so confident until we have completed the entire process*. *So*…*but*, *I mean again*, *I suppose you will need to learn this*, *you know*, *along the process" (P4*, *receiver*, *institutional lead)*

After the receivers learned the whole process of RLOs, they successfully applied the newly learned knowledge in their own institutions. They also extended the application of the RLOs concepts into other projects beyond the ACoRD project.

*“I think besides the Triumphs Projects (a project using RLOs to guide type 2 diabetes patients making decision for insulin initiation)*, *we are applying (the knowledge) to teach them this method (RLOs development)*. *I think there’s also another project on prostate cancer (a project guiding general practitioners on counsellling patients about prostate cancer screening)*.*” (P6*, *receiver*, *project manager)*

#### Subtheme 3.2: Challenges in engaging the institutions and colleagues in prioritising RLOs

As there was a lack of established eLearning technical teams in the receiver institutions, the institutional leads were concerned about the lack of expertise and resources to support the technical development of RLOs in their respective institutions.

*“I think initially when we came back from Nottingham*, *[one of the institutional leads from receivers] was quite worried…we have problems because we do not have the technical team to support us…the main part that we are worried about is the coding…” (P2*, *receiver*, *institutional lead)*

Receivers expressed that they faced challenges in applying and expanding the development of RLOs in their own institutions. The main barrier was to engaging and getting support from the higher management of HEIs.

*“…we tried to approach the higher management and explain and all that*, *but it seems that it wasn’t put as the priority*.*“ (P2*, *receiver*, *institutional lead)*

*“They do have priority in e-learning*, *but supporting the RLO is a different method*. *(P20*, *receiver*, *educator)"*

To apply the RLO concepts on a bigger scale, receivers stated that they lacked financial resources and technical support from their institutions.

*“…it really requires co-operation from the top organisation*, *financial resources on top of a technical support which is pretty much lacking in our institution at the moment…" (P21*, *receiver*, *educator)*

Besides institutional support, one of the receivers described the challenge of getting the buy-in from the staff in their institutions to support the development of RLOs.

*“I see it is in terms of the buy-in from the staff…the mentality shift will be the main challenge…whether they see the benefit of the RLO in their courses*.*” (P4*, *receiver*, *institutional lead)*

### Theme 4: Individual and organisational cultural differences

#### Subtheme 4.1: Understanding the culture of under-rating own skills and knowledge

Receivers were asked to self-report their eLearning knowledge and skills at the beginning of the ACoRD project to help the providers decide on the knowledge level that has to be transferred. Providers felt that receivers were humble and under-rated their actual level of knowledge and skills in eLearning development during the initial self-assessment. Providers were aware of the cultural difference in self-assessment among the receivers, which might influence the reporting of their baseline skills and knowledge.

*“*‥*they (Malaysian partners) might say that they have the intermediate level*, *but they might be expert actually…People tend to downgrade themselves a little bit*. *I don’t know if that’s the case I assume more or less*.*” (P7*, *provider*, *project manager)*

One of the providers expressed that self-reporting might not be a good method to assess the baseline knowledge of Malaysian partners due to cultural differences.

*“…people listed the level they are*, *what they know and what they don’t*. *And that goes back to the cultural differences*…*are people (receivers) more comfortable in indicating things that they don’t know*? *Because if they perceive this as an assessment for example*, *this might not be a good methodology*, *I guess*.*” (P8*, *provider*, *project manager)*

#### Subtheme 4.2: Difference between clan and hierarchy organisational culture

Providers noticed that receivers have a more obvious hierarchical order in their working culture where receivers often have to go through their leaders for decisions.

*“We have some differences also*, *usually in Europe especially from the country that we are coming we don’t have so obvious hierarchical order in the university*.*” (P7*, *provider*, *project manager)*

However, providers understood the working culture among the receivers, accommodating the difference in organisational culture for effective collaborations between providers and receivers.

*“And somehow the Malaysian partners*, *all three institutions somehow you assign the responsibility to the right people*, *to collaborate with the people that they have to freely without having the restriction that everything has to go through the lead partner*. *So*, *I think both the European and Malaysian partners…put common logic in place*, *take a step back in them so far…the cultural*, *the way that they used to behave*. *So*, *we can actually find a place in the middle*, *that is acceptable for the both sides so we can be collaborating in a really effective way I would say*.*” (P7*, *provider*, *project manager)*

One of the strategies was to establish constant communication between the providers and receivers at all levels and across different roles; this has helped to improve understanding and build trust between the providers and receivers.

*“…it comes down to the individuals… there is good communication at every level*, *starting from…the institution leads…down to the technical team…” (P7*, *provider*, *project manager)*

### Theme 5: Disruption of knowledge transfer during the COVID-19 pandemic

The COVID-19 pandemic happened during the ACoRD project. Two physical visits to the providers’ institutions were cancelled due to global travel restrictions. Receivers missed the opportunity to visit EU institutions to learn more about the organisation and physical setup of their eLearning units. The providers also expressed that they missed personal interactions and ‘small talks’ which occurred outside the meetings; it was through these interactions that the providers and receivers built rapport and enhanced their collaborations.

*“…We did not get to go to Stavanger… there are so many other experiences and learning that you can get from visiting different institutions and seeing the setup*, *how is it set up*? *And those are sometimes*, *the intangible things that we don’t know that we missed*, *but we missed*.*” (P11*, *receiver*, *instructional designer)*

*“…you’re going to have all those small talks*…, *within the meeting*, *outside of the meeting*… *that somehow boost all the activities to go further*. *So I*, *I find it that a big barrier that those meetings couldn’t*… *take place*, *and I don’t believe that they can*, *you know*, *easily replicated by an online meeting*.*” (P7*, *provider*, *project manager)*

Although there was a delay in the progress of RLO development and implementation during the pandemic, the knowledge transfer process continued with the prompt support given by the providers. Both parties communicated and worked together via emails and online meetings.

*“Of course*, *it is a huge barrier*, *I think*, *to be so far away from each other and meeting now and then online*. *I think that always is a challenge*. *But I think it’s going pretty well*. *There are these normal ways of sharing ideas*, *just talking*, *discussing and sharing work on paper or online works*.*” (P1*, *provider*, *institutional lead)*

*“I think in terms of the support*, *I think we’ve got all the support that we need*, *in a timely manner…I’m referring to the [providers’ technologists]*, *he’s been very helpful… and yeah*, *the support is actually tremendous*. *They actually get back to us quite*, *quite fast*, *and I think the fact that we have our monthly meeting helps us as well*, *to align*, *and also to identify*, *and to discuss sort of support that we need and address it accordingly*.*” (P10*, *receiver*, *instructional designer)*

The RLOs were planned to be incorporated into the existing medical and bioscience curricula. However, due to the COVID-19 pandemic, some of the curricula were changed, leading to a delay in the implementation of the RLOs. Educators from the receiver group had to improvise and explore other strategies to implement the RLOs.

*“…we have planned to incorporate in the curriculum…because of the COVID*, *the curriculum change*… *and we have to like adjust to see when we can put that in…that’s why some of the RLOs are still not been incorporated yet*.*” (P2*, *receiver*, *institutional lead)*

## Discussion

Our study highlights the challenges both knowledge providers and receivers faced in the knowledge transfer process on digital learning objects development. Both interpersonal and inter-organisational challenges were identified: mismatched expectations, learning new knowledge beyond own professions, barriers to applying knowledge in new environments, cultural differences, and the COVID-19 pandemic. Building upon the knowledge management perspective, we proposed strategies to foster knowledge transfer in the context of building the capacity for digital learning development in HEIs.

Prior experience and related knowledge are essential factors influencing the knowledge transfer process between organisations [6]. Our study uncovers the mismatched scope and depth of knowledge to be transferred between the providers and receivers, highlighting the importance of baseline knowledge and needs assessment in a knowledge transfer process. Identifying learning needs is an essential part of educational planning, learners’ progress assessment, and allowing individual feedback [17]. To achieve successful knowledge transfer in digital learning development, a comprehensive assessment of individual and organizational needs and gaps in knowledge components is needed to plan the knowledge transfer program. Using Delphi surveys and semi-structured interviews, a systematic approach can be conducted to identify organisational knowledge needs [18]. Our study asserts that baseline knowledge and needs assessment may not be sufficient, and continuous assessment is required along the process of knowledge transfer. Our findings suggest that continuous bidirectional feedback could be a strategy to harmonise the expectations between two parties. This feedback method has been well-recognised in educational literature to advance students’ learning [19] and can be applied in knowledge transfer. Bidirectional feedback between receivers and providers would reduce the expectation gaps between organisations and close the feedback loops by making adjustments in the knowledge transfer process [20].

Our study demonstrates the challenges of both technologists and academics in learning new skills out of the scope of their professional knowledge. To succeed in interprofessional collaboration, professionals need to build upon the knowledge of their professions and integrate the knowledge of other professionals into practice [21, 22]. This attitude is essential for interprofessional collaboration in digital learning development as it requires intersections and teamwork between content authors, instructional designers, and technical developers [9]. A successful transdisciplinary knowledge transfer and collaboration often require an exchange of knowledge, skills, and expertise beyond their own discipline boundaries [23]. In our study, it is interesting to find out that there was bidirectional knowledge transfer where technologists from the provider team gained tacit knowledge in teaching and research via the experience of training the receivers and being involved in research activities. Often, knowledge transfer has been treated as a one-way linear process where receivers learn the knowledge, skills, and technology from the providers, and providers only learn from the receivers’ experience of implementing the knowledge in a new environment [5]. Our experience of bidirectional knowledge transfer in digital learning development suggests a need to create a positive and conducive environment for both providers and receivers to interact and foster mutual knowledge creation. Formal and informal interactions and relationships, especially between partners from different geographical regions, promote shared understanding and inter-organisational tacit knowledge flows [24].

Knowledge application is the most significant yet challenging process in knowledge transfer [25]. Our study highlights both personal and organisational barriers to applying newly learned knowledge in Malaysian HEIs. Our study suggests that practical experience in digital learning materials development is required before the receivers can transform and apply the newly learned knowledge in their institutions. We propose that a training course design that follows the learners’ progress would assist the transfer of tacit knowledge, including the strategy of learning by doing to enhance learners’ knowledge absorption and dissemination [26]. Another challenge of knowledge application in our study is engaging people and organisational management to keep the digital learning innovation beyond the project’s boundaries. Often, practical and political efforts are required to create common interests to share and transform knowledge in a new environment [27]. Strategies to successfully apply the newly learned knowledge in HEIs are pragmatic, requiring dissemination of the benefits of the innovation and negotiation to achieve shared interest and aligned goals.

The inter-organisational knowledge transfer occurs in the social interaction process between individuals and organisations [8]. Cultural difference between HEIs with wide geographical distance was a challenge faced in the knowledge transfer process. During the self-evaluation of baseline knowledge, we observed the difference in Malaysian partners who under-reported their baseline knowledge. Our study echoes previous studies that Asians have a greater tendency to self-effacement during self-evaluation due to the modesty norm in Eastern culture [28, 29]. Hence, we suggest a multifaceted evaluation method could be employed to assess the knowledge needs to reduce such discrepancy in self-assessment. Besides that, the difference in clan and hierarchy organisational culture between HEIs was highlighted in our study. Malaysia’s organisational management trend is mainly hierarchical and collectivistic culture, emphasising respect, trust, and personal relationships [30]. Literature has shown that clan organisational culture has a more positive influence on tacit knowledge sharing than hierarchy organisational culture [31]. However, the effect of hierarchical organisational culture on the knowledge transfer process was not obvious in our study. This may be because of the participation in a capacity-building project like ACoRD where members are inherently collaborative in a non-hierarchical manner. Acknowledgment and mutual understanding of cultural differences are essential in the East-West inter-organisational collaboration. Cultural difference is often viewed as a double-edged sword in cross-national collaboration. However, mutual understanding can maximize the positives to enhance knowledge transfer [32].

The easiness of transferring knowledge is influenced by the accessibility and relationship between the providers and receivers [33]. Our study reveals a relational barrier in knowledge transfer during the COVID-19 pandemic between the providers and receivers due to limited informal communication and social interaction. Also, our results show a disruption in tacit knowledge transfer when receivers applied the knowledge of implementing RLOs in the curricula during the pandemic. With the lack of informal knowledge sharing, it is more challenging for tacit knowledge transfer than before COVID-19 [34]. In our study, the knowledge-related barrier was less evident during the pandemic because the knowledge transfer process continued with the adoption of communication technologies. This is consistent with the phenomenon described by Burak et al. [33], where the relational barrier increased in knowledge transfer during the pandemic, but not the knowledge-related barrier. The use of communication technologies might reduce the geographical barrier to explicit knowledge transfer [35].

### Strategies for international knowledge transfer in digital learning development

There are several key lessons that we have learned from this knowledge transfer process in this ACoRD project. We suggest several strategies to enhance future interprofessional and cross-national knowledge transfer, especially among HEIs in digital learning materials development.

- Apply a systematic and continuous needs assessment for both knowledge receivers and providers. This could be achieved via timely bidirectional feedback to address expectations and challenges. We suggest carrying out a needs analysis before knowledge transfer activities to tailor the training as closely as possible to receivers’ needs.
- Create a conducive environment for both knowledge providers and receivers to acquire new knowledge beyond their profession.
- Develop pragmatic strategies in cascading the new knowledge to the knowledge receivers’ organisations
- Acknowledge and address cultural differences between HEIs and optimise its positives to enhance the knowledge transfer process.

## Conclusion

This study highlights the challenges and provides strategies for knowledge transfer across professional and geographical boundaries in HEIs for capacity building in digital learning development. The findings from this study could benefit future international collaborations and knowledge transfer among HEIs.

## Supporting information

S1 File Full topic guide to explore knowledge transfer between HEIs. (DOCX)
