# Supplementary material for: Knowledge transfer of eLearning objects: Lessons learned from an intercontinental capacity building project
Source: PLoS One. 2022 Sep 20;17(9):e0274771. doi: 10.1371/journal.pone.0274771 (PMC9488788; doi:10.1371/journal.pone.0274771)
Supplement: S1 File — (DOCX) [file pone.0274771.s001.docx]

**S1 File: Full topic guide to explore knowledge transfer between HEIs.**

**Focus Group Discussion (FGD) Round 1: Aims, Storyboarding, Population**

Round 1: FGD for providers (EU partners)

| **No** | **Question** |
| --- | --- |
| **1.** | **The overall experience of knowledge transfer** |
|  | Can you tell me, as a whole, your experience as a partner in the ACoRD project so far?  *Probe:*   - *What do you see as your role in this project?* - *What have you achieved so far in transferring knowledge to the Malaysian partner institutions?* |
| **2.** | **Awareness** |
|  | Do you encounter any problem when sharing your knowledge with Malaysian partners?  *Probe:*   - *What are the challenges?* - *Is there any way that makes the knowledge transfer easier?*   *Let us talk about our first project event which is the stakeholder and train-the-trainer workshop in UoN.*   - *What do you think about the workshop?* - *Is there anything that* ***should*** *have been included in the first workshop?* - *Is there anything that* ***should not*** *have been included in the first workshop?* - *What do you think are the gaps in the Malaysian partners’ knowledge about RLOs?* |
| **3.** | **Acquisition** |
|  | *(not applicable for the EU partners)* |
| **4.** | **Transformation, Association, Application** |
|  | 1. You have seen the Malaysian partners conducting the workshop in June.  - Do you have any comments on how they apply the knowledge that they have learned? - Is there anything that they can improve?  1. You have seen the Malaysian partners creating the storyboards and specifications with their students and educators.  - What are your comments on how they conducted the storyboarding and specification in their institutions? - Is that anything that they can improve?   *Probe:*  *Do you think the Malaysian partners were able to translate what they have learned into training the educators and creating storyboards/specifications?*   - *If yes, why?* - *If no, why not?* |
| **5.** | **Knowledge externalization/feedback** |
|  | Do you think there is enough opportunity for you to receive feedback from the Malaysian partners?   - - Can you tell us about how it happened?   - What was the feedback so far?   - How useful the feedback? |
|  | *Prompts:*   - International collaboration - Gap of background knowledge - Goals and expectation - Institutional capacity - Institutional culture - Trust - Communication - Past experience - Leadership - Funding and resources - Time - Competing priority |
| **End** | **Thank you for your time and input.** |

Round 1: FGD for receivers (Malaysian partners)

| **No** | **Question** |
| --- | --- |
| **1** | **Overall experience of knowledge transfer** |
|  | What do you think about the process of transferring the knowledge to the Malaysian partners in this project?  *Probe:*   - What do you see as your role in this project? - What have you achieved so far in receiving/adopting the knowledge from the EU partner institutions? |
| **2** | **Awareness** |
|  | Do you encounter any problem when receiving knowledge from the EU partners?  *Probe:*   - *What are the challenges?* - *Is there any way that makes the knowledge transfer easier?*   *Let us talk about our first project event which is the stakeholder and train-the-trainer workshop in UoN.*   - *What do you think about the workshop?* - *What do you think are the gaps in the Malaysian partners’ knowledge about RLOs?* - *Is there anything that you* ***would like to be included*** *in the first workshop?* - *Is there anything that* ***should not*** *have been included in the first workshop?* |
| **3** | **Acquisition** |
|  | How well do you think you have learned from training sessions?  *Probe:*   - *What are the challenges?* - *Is there any way that makes the knowledge transfer easier?*   *Probe:*   - *How well do you think you have learned from the train-the-trainer workshop in Jan (UoN)?*    - *Pedagogical method of RLOs*   - *Tools (storyboard, specification)* - *How well do you think you have learned from the Hands-on Technical Training event workshop in June (UM)?* |
| **3** | **Transformation, Association and application** |
|  | What do you think about translating and applying what you have learnt into your own institution?  *Probe:*  *A) About Malaysian partners conducting RLOs co-creation workshop in June 2019 (UM)*   - *What do think about the RLOs Co-creation workshop run by you (Malaysian partners)?* - *Do you think that you were able to translate what they have learned into conducting the workshop?*   - *If yes, why?*   - *If not, why not? Give examples for each* - *What are the challenges that you faced?*   *B) About Malaysian partners doing the storyboard and specification*  *You (Malaysian partners) have conducted internal workshops on storyboarding with the students and educators.*   - - *What do think about the internal workshops run by you (Malaysian partners)?*   - *Do you think you were able to translate the knowledge and resource (from UoN) into conducting the storyboard workshop with the students and educators?*      - *If yes, why?*     - *If not, why not?*   - *Do you think you were able to translate the knowledge and resource into conducting specification workshops with the educators?*     - *If yes, why?*     - *If not, why not?*   - *What are the challenges that you faced?* |
| **4.** | **Knowledge externalization/feedback** |
|  | Do you give feedback to UoN in the process of the knowledge transfer in developing RLOs?   - How was the feedback process to far? - Are they useful? |
|  | *Prompts:*   - International collaboration - Gap of background knowledge - Goals and expectation - Institutional capacity - Institutional culture - Trust - Communication - Past experience - Leadership - Funding and resources - Time - Competing priority |
| **End** | **Thank you for your time and input.** |

**Focus Group Discussion (FGD) Round 2: Implementation, Release, Evaluation**

Round 2: FGD for providers (EU partners)

| **No** | **Question** |
| --- | --- |
| **1.** | **Roles in knowledge transfer** |
|  | At this current stage, the Malaysian partners have done the technical development, incorporated the RLOs into the curriculum and evaluated the RLOs.   - What is your role in supporting the Malaysian partners through the process? - What have you achieved so far in transfering the knowledge to the Malaysian partners? |
| **2.** | **Awareness** |
|  | Do you encounter any problem when sharing your knowledge with Malaysian partners?   - Technical development - Incorporating RLOs into curriculum - RLOs evalution   *Probe:*   - *What are the challenges?* - *Is there any way that makes the knowledge transfer easier?* |
| **3.** | **Acquisition** |
|  | *(not applicable for the EU partners)* |
| **4.** | **Transformation, Association, Application** |
|  | You have seen the Malaysian partners’ progress in:   - Technical development - Incorporating RLOs into curriculum - RLOs evalution   Do you think the Malaysian partners have applied the knowledge that they have learned?   - If yes, how they have applied? Give examples - If no, why do you think so? Give reasons |
| **5.** | **Knowledge externalization/feedback** |
|  | Do you think there is enough opportunity for you to receive feedback from the Malaysian partners in the process of technical development, incorporating and evaluating the RLOs?   - - Can you tell us about how it happened?   - What was the feedback so far?   - How useful the feedback?   Do you think the Malaysian partners have transferred some knowledge to the EU partners?   - If yes, what is the knowledge that you have received? Give examples |
|  | *Prompts and areas to explore further from the first FGD.*   - The effect of COVID-19 pandemic on knowledge transfer between EU and Malaysia - Interaction between technical team and academia - Cultural difference – how hierarchical order among Malaysian partners affect the knowledge transfer process |
| **End** | **Thank you for your time and input.** |

Round 2: FGD for receivers (Malaysian partners)

| **No** | **Question** |
| --- | --- |
| **1** | **Roles in knowledge transfer** |
|  | At this stage, we have done the technical development, incorporate the RLOs in the curriculum and evaluate the RLOs.   - What is your role at this stage of project? |
| **2** | **Awareness** |
|  | Can you share your learning experience on:   - How to develop the technical aspect of RLOs - Incorporate the RLOs into curriculum - Evaluate the RLOs   Probes:  - What are the challenges?  - How you solve them?  - Did you seek help/advice from EU partners?   - If yes, how is the advice/help of EU partners - If not, why not? |
| **3** | **Acquisition** |
|  | What do you think you have learned from the EU partners in term of:   1. Technical development 2. Incorporate the RLOs into the curriculum 3. Evaluation of RLOs |
| **4** | **Transformation, Association and application** |
|  | Since you have learned from EU partners about RLOs, have you applied what you have learnt into your own/other institution?  Probe:   - If yes, how have you applied? Give examples - If no, why not? Give reasons - What are the challenges that you faced? |
| **4.** | **Knowledge externalization/feedback** |
|  | Do you give feedback to EU partners on how they support the process of developing, incorporating and evaluating the RLOs?   - Do you find any challenges in giving feedback to the EU partners? - How was the feedback process to far? - Are they useful?   Do you think you have shared some knowledge to the EU partners?   - If yes, what knowledge? Give examples |
|  | *Prompts and areas to explore further from the first FGD)*   - The effect of COVID-19 pandemic on knowledge transfer between EU and Malaysia - Cultural difference – hierarchical order among Malaysia partners - Interaction between technical team and academia |
| **End** | **Thank you for your time and input.** |
